# BIMALLEOLAR ANKLE FRACTURE: A SIMPLE FRACTURE?

**DOI:** 10.1590/1413-785220172501166234

**Published:** 2017

**Authors:** JUNJI MILLER FUKUYAMA, ROBINSON ESTEVES SANTOS PIRES, PEDRO JOSÉ LABRONICI, JOSÉ OCTÁVIO SOARES HUNGRIA, RODRIGO LOPES DECUSATI

**Affiliations:** 1. Hospital Geral de Vila Penteado, São Paulo, SP, Brazil.; 2. Universidade Federal de Minas Gerais, Hospital das Clínicas e Hospital Felício Rocho, Belo Horizonte, MG, Brazil.; 3. Universidade Federal Fluminense, Rio de Janeiro, RJ, Brazil.; 4. Santa Casa de São Paulo, São Paulo, SP, Brazil.; 5. Hospital Geral do Campo Limpo, São Paulo, SP, Brazl.

**Keywords:** Ankle, Ankle fractures, Ligaments, Magnetic resonance imaging

## Abstract

**Objective:**

To evaluate the frequency of deltoid ligament injury in bimalleolar supination-external rotation type fractures and whether there is a correlation between the size of the fractured medial malleolus and deltoid ligament injury***.***

**Methods:**

Twenty six consecutive patients underwent magnetic resonance exams after clinical and radiographic diagnosis of bimalleolar supination-external rotation type ankle fractures***.***

**Results:**

Thirteen patients (50%) presented deltoid ligament injury associated to bimalleolar ankle fracture. Partial injury was present in seven (26.9%) patients and total injury in six (23.1%). Regarding medial fragment size, the average was 2.88 cm in the absence of deltoid ligament injury. Partial injuries presented 1.93 cm and total 2.1 cm on average***.***

**Conclusion:**

Deltoid ligament injury was present in 50% of bimalleolar ankle fractures. Smaller medial malleolus fragments, especially concerning the anterior colliculus, presented greater association with partial deltoid ligament injuries. ***Level of Evidence IV, Cross Sectional Study.***

## RESUMO

**Objetivo:**

***Avaliar a frequência da lesão do ligamento deltóide nas fraturas bimaleolares do tornozelo tipo supinação-rotação externa e se existe correlação entre o tamanho do fragmento do maléolo medial fraturado e o tipo de lesão do ligamento deltóide.***

**Métodos:**

***Vinte e seis pacientes consecutivos com diagnóstico clínico e radiográfico de fratura bimaleolar do tornozelo tipo supinação-rotação externa (Lauge-Hansen), foram submetidos ao exame de ressonância magnética do tornozelo para avaliar a presença de lesão ligamentar associada à fratura bimaleolar.***

**Resultados:**

***Treze pacientes (50%) apresentaram lesão do ligamento deltóide associada à fratura bimaleolar do tornozelo, sendo sete (26,9%) lesões parciais e seis (23,1%) totais. Com relação ao tamanho do fragmento do maléolo medial, na ausência de lesão do ligamento deltóide, o tamanho médio foi de 2,88 cm. Nas lesões parciais, a média foi de 1,93 cm e, nas lesões totais do ligamento deltóide, de 2,1 cm.***

**Conclusões:**

*A lesão do ligamento deltóide esteve presente em metade das fraturas bimaleolares do tornozelo do tipo supinação-rotação externa. Fragmentos menores do maléolo medial, especialmente do colículo anterior, tem maior associação com lesões parciais do ligamento deltóide.* ***Nível de Evidência IV, Estudo Transversal.***

## INTRODUCTION

Ankle fractures are among the most common injuries treated by orthopedic surgeons.

Typically, ankle fractures result from low-energy rotational traumas. But as numbers of traffic accidents have increased, the severity of the fractures and trauma energy have grown steadily.^1^ ^-^ ^5^

Stability of the ankle joint is provided by the medial and lateral malleoli and their respective ligaments.

Careful identification of these injuries and their treatments involve not only recognition of bone injuries, but also identification of damage to the soft tissue and ligaments.

Tornetta^6^ described the importance of assessing the ankle joint through traditional x-ray parameters and stated that the ankle joint must not be assessed as only a bony component, but rather an osteoligamentous joint surrounded by soft tissue. These lateral and medial ligament complexes have an important relationship with the pathophysiology of ankle fractures and with the therapeutic approach.

The deltoid ligament is the primary stabilizer of the ankle. Its superficial and deep tracts can rupture as a result of rotational trauma, leading to instability and chronic pain in this joint.^7^

The authors of this study hypothesize that magnetic resonance imaging (MRI) can provide important information for identifying ligament injuries associated with bimalleolar fractures of the ankle joint such as supination-external rotation according to the Lauge-Hansen classification.

The aim of this study was to obtain MRI from patients with supination-external rotation type bimalleolar ankle fractures in order to assess the frequency of concomitant deltoid ligament injuries and the relationship between the size of the fractured medial malleolus fragment and the type of injury to the deltoid ligament.

## METHODS

The present study was approved by the Institutional Review Board of the main institution under the number 15632813.9.0000.5149 and was conducted in accordance with the standards of the Helsinki Declaration.

The sample consisted of 26 consecutive patients treated at a general hospital with a clinical and radiographic diagnosis of bimalleolar fracture of the ankle, Lauge-Hansen supination-external type; MR imaging (using a Philips Intera 1.5 tesla device) was obtained for the affected joint.

The resulting imaging was analyzed by a radiologist specializing in MRI and a member of the Brazilian College of Radiology, as well as a physician belonging to the Brazilian Society of Orthopedics and Traumatology and the Brazilian Society of Orthopedic Trauma.

The inclusion criteria were: adult patients (17 years or older) with supination-external rotation type bimalleolar fracture of the ankle who read and signed the terms of free and informed consent and agreed to be included in the study.

The exclusion criteria were: skeletally immature patients, patients who refused to sign the terms of consent, and patients with other ankle fracture patterns than those mentioned in the inclusion criteria.

Sample: Of the 26 patients, 16 (61.54%) were male and 10 (38.46%) were female. Eleven patients (42.32%) had trauma on the left side and 15 (57.69%) on the right side. 

The type of trauma was car accident in six patients (23.1%), pedestrian hit by a vehicle in two patients (7.7%), fall from height in four patients (15.4%), trip-and-fall in 13 patients (50%), and sports trauma in one patient (3.8%).

Patient age ranged from 19 to 50 years, with a mean of 32.3.

A descriptive analysis was performed of the data and 95% confidence intervals (CI) were constructed for the proportions sex, side, presence of injury to the deltoid ligament, presence of total deltoid ligament rupture, and presence of partial deltoid ligament rupture. Since the sample size was less than 30 and the central limit theorem could not be used, we used exact calculation of the confidence interval based on binomial distribution, the interval of which is generally not symmetrical in relation to the estimated proportion. We also constructed 95% confidence intervals for the mean age and mean size of the fragment of the medial malleolus. To calculate this latter interval, because the sample was smaller than 30 and the central limit theorem again could not be used, we tested the adhesion of the variables age and size of the fragment of the medial malleolus and normal distribution using the Anderson Darling test.^8^

Non-parametric analysis of variance was conducted using the Kruskal-Wallis test to test at a 5% significance level whether the size of the fragment of the medial malleolus is identical in all three groups (no injury to the deltoid ligament, partial rupture of the deltoid ligament, and total rupture of the deltoid ligament), making contrasts by pairs to detect which differences were significant.^9^

The hypothesis that the average size of the medial malleolus fragment in patients with total ligament rupture was no greater than 2.8 cm was also tested against the alternative hypothesis that the fragment in this group was greater than 2.8 cm at 5% significance using the non-parametric Wilcoxon signed-rank test. We also tested the hypothesis that the average size of the medial malleolus fragment in patients with partial ligament injury was at least 1.7 cm against the alternative hypothesis that the fragment was smaller than 1.7 cm, according to the parameters standardized by Tornetta.^6^

Since the hypothesis tests were performed at a 5% significance level, hypotheses with descriptive levels below 0.05 were also discarded.

## RESULTS

Thirteen patients (50%) exhibited injury to the deltoid ligament (CI=[29.93; 70.07]%), seven (26.9%) had partial rupture of this ligament (CI =[11.57; 47.79]%), and seven (23.1%) had total rupture of this ligament (CI = [8.97; 36.07]%).

The size of the fragment of the medial malleolus ranged from 1.6 cm to 3.3 cm with a mean of 2.6 cm and standard error of 0.101 cm. We tested adherence to the size distribution of the medial malleolus fragment to the normal distribution, and this hypothesis was not rejected (P = 0.061). Consequently, the CI for the medial malleolus fragment was [2.362; 2.777] cm.

**Figure 1:**
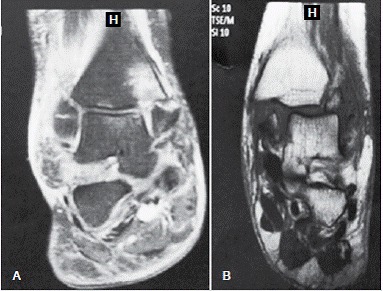
(A) Magnetic resonance imaging of the ankle in coronal plane showing a Weber B fracture, supination-external rotation stage 4, with fragment of the medial malleolus containing a small deviation and integrity of the deltoid ligament. (B) Magnetic resonance imaging of the ankle in coronal plane showing a Weber B fracture, supination-external rotation stage 4, with medial malleolus fragment 2.3 cm in size and total rupture of the deltoid ligament.

Figure 1 illustrates the magnetic resonance imaging for two patients with fractures of the medial malleolus.

The Kruskal-Wallis test was used to evaluate the hypothesis that the size of the medial malleolus fragment was identical in all three groups (no injury to the deltoid ligament, partial rupture of the deltoid ligament, and total rupture of the deltoid ligament), and this hypothesis was rejected (P = 0.001). Contrasts showed at 5% significance that the size of the medial malleolus fragment differs (is smaller) when the rupture is partial when compared to the respective fragment sizes and other injury types (total rupture, no injury). It was also concluded that there was no significant difference in the size of the medial malleolus fragment when there was no injury to the deltoid ligament and when a total rupture was present.

**Figure 2:**
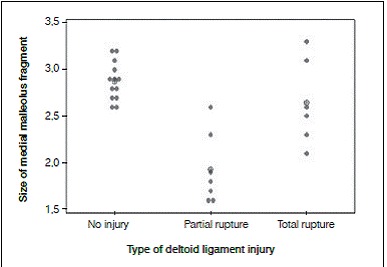
Graph of individual values for medial malleolus fragment size and mean (circle with a cross) for each group.

Table 1 and Figure 2 present the results.

**Table 1:** Measurements of medial malleolus fragment (in cm), mean, and standard error (SE).

Injury	Absent	Partial	Total
	2.9	1.6	2.3
	2.8	2.6	3.3
	3.2	1.8	2.5
	2.9	1.6	2.6
	2.9	2.3	3.1
	2.7	1.7	2.1
	2.6	1.9	
	2.8		
	3.2		
	2.6		
	2.7		
	3.1		
	3.0		
Mean	2.88	1.93	2.65
SE	0.06	0.14	0.19

SE: Standard Error

The non-parametric Wilcoxon signed-rank test at 5% significance was used to test the hypothesis that the mean size of the medial malleolus fragment in patients with total ligament rupture was no more than 2.8 cm, and this hypothesis was not rejected (P = 0.799). The hypothesis that the mean medial malleolus fragment with partial ligament rupture was at least 1.7 cm was also tested at 5% significance, and this hypothesis was also not rejected (P = 0.929).

## DISCUSSION

Fractures classified by Lauge-Hansen as supination-external rotation type are the most prevalent malleolar fractures, accounting for 40-75% of ankle fractures.^4^ ^,^ ^5^ ^,^ ^10^In this fracture pattern, the injury begins in the lateral component, and may progress to the medial side depending on the energy of the trauma. The fourth stage of the injury involves the deltoid ligament and/or fracture of the medial malleolus.

The superficial deltoid ligament, which originates in the anterior colliculus, does not contribute greatly to ankle stability.^11^

**Figure 3:**
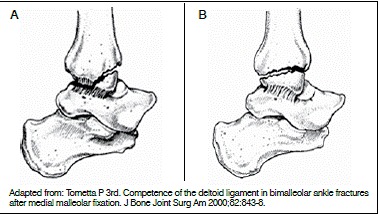
(A) anterior colliculus fracture with small fragment and continuous injury of the deep fibers of the deltoid ligament (partial tear: posterior and deep portion); (B) medial malleolus fracture with the fracture line passing through all the collicular surfaces, causing medial instability in the ankle but without injury to the deltoid ligament.

Recently, the ligament injuries associated with anterior colliculus fracture of the medial malleolus have been described.^6^ ^,^ ^11^ ^-^ ^17^ In this pattern of medial malleolus fracture, we find fracture of the anterior colliculus with the posterior colliculus intact. This allows the energy to pass through the posterior portion of the deltoid ligament and causes a partial rupture in this ligament. (Figure 3)

Therefore, the size of the medial fragment injury is intimately linked to the competence and integrity of the deltoid ligament. Tornetta^6^ found that fractures of the medial malleolus greater than 2.8 cm in length (supracollicular fractures) have intact ligaments, while malleolar fractures smaller than 1.7 cm (fracture of the anterior colliculus or intercollicular fractures) compromise the competence of the deltoid ligament.

In the present study, seven patients presented partial rupture of the deltoid ligament, predominantly involving the deep portion. These data resemble the findings by Tornetta,^6^ who found 26% incompetence of the deltoid ligament.

Based on the data presented regarding the average size of the medial malleolus fragment and injury to the deltoid ligament demonstrated in the MRI, we found a relationship between the size of the medial malleolus fragment and the rupture of the deltoid ligament.

This analysis found similar average values in relation to the size of the medial malleolus fragment, and showed that fractures with larger fragments also tended to feature an intact deltoid ligament. Fractures with smaller fragments of the medial malleolus showed partial rupture of the deltoid ligament with integrity in the superficial portion (usually still inserted in the anterior colliculus) and rupture of the deep portion. Total rupture of the deltoid ligament was found in patients with bone fragments with an average size of 2.6 cm.

Limitations of this study include a relatively small sample size (26 patients), lack of pre- and post-hoc power analysis, and lack of correlation between the presence of deltoid ligament injury associated with bimalleolar fracture and treatment or prognosis. MRI is still a relatively expensive examination and is not available for most emergency medical services in developing countries.

Among the positive points of this study, the authors confirmed the hypothesis that MRI is valuable in diagnosing ligament injuries of the ankle associated with bimalleolar fractures, confirming the findings by Tornetta.^6^ It is essential to stress the importance of early identification of injuries to the deltoid ligament concomitant with ankle fractures, an injury that seems to be more frequent than previously thought. The authors were unable to find any studies in Portuguese published at the time of this writing demonstrating this common association.

## CONCLUSION

In Lauge-Hansen type supination-external rotation bimalleolar fractures of the ankle, the deltoid ligament was also ruptured in half of cases. When the deltoid ligament rupture was partial, the medial malleolus fragment was smaller (fracture only in the anterior colliculus) than when the ligament was completely ruptured or when there was no injury.

There was no significant difference in the size of the medial malleolus fragment when there was no injury to the deltoid ligament and when the rupture was total.

Future studies with more meaningful sample sizes and a cost-benefit analysis of introducing MRI into the diagnostic arsenal of emergency services for ankle fractures are needed to validate the hypothesis that bimalleolar fractures, which are considered relatively easy to treat, may present associated ligament injuries that can lead to instability and chronic pain in the ankle if not treated properly.
